# Additive Fabrication of Polyaniline and Carbon-Based Composites for Energy Storage

**DOI:** 10.3390/polym16233369

**Published:** 2024-11-29

**Authors:** Niwat Hemha, Jessada Khajonrit, Wiwat Nuansing

**Affiliations:** 1 School of Physics, Institute of Science, Suranaree University of Technology, Nakhon Ratchasima 30000, Thailand; niwat6842@gmail.com; 2 Department of Science and Mathematics, Faculty of Science and Health Technology, Kalasin University, Kalasin 46000, Thailand; ex_phys@hotmail.com; 3 Center of Excellence on Advanced Functional Materials, Suranaree University of Technology, Nakhon Ratchasima 30000, Thailand

**Keywords:** rGO, supercapacitor, 3D printing, direct ink writing (DIW), cellulose acetate (CA), polyaniline (PANI)

## Abstract

The growing demand for efficient energy storage systems, particularly in portable electronics and electric vehicles, has led to increased interest in supercapacitors, which offer high power density, rapid charge/discharge rates, and long cycle life. However, improving their energy density without compromising performance remains a challenge. In this study, we developed novel 3D-printed reduced graphene oxide (rGO) electrodes coated with polyaniline (PANI) to enhance their electrochemical properties. The rGO 3D-printed electrodes were fabricated using direct ink writing (DIW), which allowed precise control over thickness, ranging from 4 to 24 layers. A unique ink formulation was optimized for the printing process, consisting of rGO, cellulose acetate (CA) as a binder, and acetone as a solvent. The PANI coating was applied via chemical oxidative polymerization (COP) with up to five deposition cycles. Electrochemical testing, including cyclic voltammetry (CV), galvanostatic charge/discharge (GCD), and electrochemical impedance spectroscopy (EIS), revealed that 12-layer electrodes with three PANI deposition cycles achieved the highest areal capacitance of 84.32 mF/cm^2^. While thicker electrodes (16 layers and beyond) experienced diminished performance due to ion diffusion limitations, the composite electrodes demonstrated excellent cycling stability, retaining over 80% of their initial capacitance after 1500 cycles. This work demonstrates the potential of 3D-printed PANI/rGO electrodes for scalable, high-performance supercapacitors with customizable architectures.

## 1. Introduction

The growing demand for efficient energy storage systems, particularly in portable electronics, electric vehicles, and renewable energy technologies, has fueled interest in supercapacitors, which offer high power density, rapid charge/discharge rates, and long cycle life. Among the various types of energy storage technologies, supercapacitors have garnered significant attention due to these unique advantages, making them a promising solution that bridges the gap between traditional capacitors and batteries [1,2,3,4,5,6]. However, despite their potential, achieving both high energy density and power density in supercapacitors remains a critical challenge, driving extensive research into advanced materials and innovative fabrication techniques to overcome these limitations [7,8,9].

One of the most promising materials for supercapacitor electrodes is reduced graphene oxide (rGO). rGO stands out due to its exceptional combination of high surface area, excellent electrical conductivity, and mechanical flexibility [10,11,12,13]. The two-dimensional structure of rGO facilitates the accumulation of electric charge, while its conductive network promotes efficient electron transport during charging and discharging processes [14,15,16,17]. However, the electrochemical performance of rGO requires enhancement through the integration of additional materials.

In this regard, polyaniline (PANI), a conductive polymer with pseudocapacitive properties, has emerged as an attractive complementary material for improving the capacitance of supercapacitor electrodes. PANI undergoes fast and reversible redox reactions, contributing to high specific capacitance [18,19,20,21,22,23,24,25]. This makes it a highly suitable candidate for supercapacitors. Applying a PANI coating to rGO allows the integration of rGO’s double-layer capacitance with PANI’s pseudocapacitance, leading to a synergistic effect that markedly improves energy storage capabilities [26,27,28,29].

While traditional fabrication methods for supercapacitor electrodes are limited in two-dimensional designs [30], direct ink writing (DIW) 3D printing offers a novel and versatile approach for fabricating electrodes with customizable architectures [31,32,33]. DIW enables the precise deposition of electrode materials in complex geometries, allowing for optimization of critical factors such as electrode thickness, porosity, and surface area [34,35,36]. These factors are essential for maximizing energy storage performance. Furthermore, DIW technology allows for layer-by-layer printing, which provides precise control over the electrode structure and dimensions, ultimately enabling the fine-tuning of electrochemical properties.

The integration of 3D printing technology into the fabrication of energy storage devices represents a significant advancement in the field. Three-dimensional printing allows for the creation of intricate, complex structures with minimal waste compared to traditional manufacturing methods like Computer Numerical Control (CNC) machining and molding [37,38]. For example, Chuan Yi Foo et al. fabricated a solid-state supercapacitor using a printable graphene-based conductive filament, demonstrating impressive specific capacitance values of 98.37 F/g [39]. Similarly, Yao Bin et al. investigated 3D-printed graphene aerogel/MnO_2_ electrodes, achieving high areal capacitance values of 44.13 F/cm^2^, showcasing the potential of 3D-printed electrodes for energy storage [40].

This study aims to develop 3D-printed rGO electrodes coated with PANI for application in high-performance supercapacitors. A novel ink formulation, consisting of rGO, cellulose acetate as a binder, and acetone as a solvent, was optimized for use in the DIW process. To the best of our knowledge, this ink formulation has not been previously investigated, making it a unique contribution to the field. The electrodes were printed with varying layer thicknesses, and subsequently coated with PANI through a chemical oxidative polymerization (COP) technique. Multiple deposition cycles were applied to control the thickness of the PANI film. The electrochemical performance of the PANI/rGO 3D-printed electrodes was evaluated using cyclic voltammetry (CV), galvanostatic charge/discharge (GCD), and electrochemical impedance spectroscopy (EIS). The focus of the study was to assess how variations in electrode thickness and PANI coating cycles affected the capacitance and charge transfer resistance of the electrodes.

## 2. Materials and Methods

### 2.1. Materials

Reduced graphene oxide (rGO) was purchased from Haydale Technology (Bangkok, Thailand). Cellulose acetate (99.99%, average Mn = 50,000) and N-Methyl-2-pyrrolidone (NMP) were obtained from Merck (St. Louis, MO, USA). Acetone (CH_3_COCH_3_, ACS reagent, ≥99.5%, Mw = 58.08 g/mol) was supplied by Labscan (Bangkok, Thailand). Potassium hydroxide (KOH, analytical reagent, >85.0%, Mw = 56.11 g/mol) was sourced from KemAus (Melbourne, VIC, Australia). Hydrochloric acid (HCl, 37%, density = 1.19 g/cm^3^ at 20 °C) was also obtained from Merck (St. Louis, MO, USA).

### 2.2. Ink Preparation

**Figure 1:**
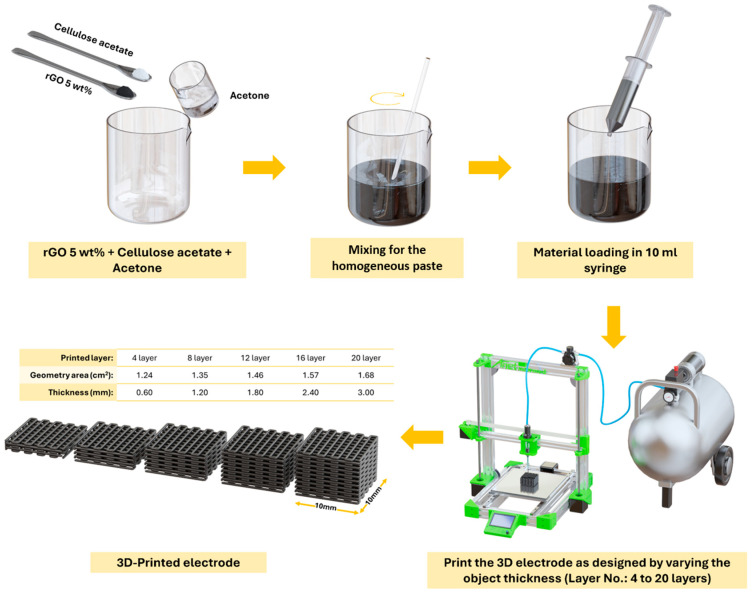
Overview of the 3D-printed supercapacitor electrode fabrication.

The ink is composed of rGO, cellulose acetate (CA), acetone, and N-methyl-2-pyrrolidone (NMP). The powdered materials, rGO and CA, are mixed in a 1:19 ratio by weight. The concentration of rGO in the ink is approximately 0.95 wt%. To prepare 10 mL of ink, 0.2 g of rGO, 3.8 g of CA, 16 g of acetone (equivalent to 20 mL, considering a density of 0.791 g/mL at 25 °C), and 1 mL of NMP are used. The rGO and CA powders are manually mixed in a single beaker. During the mixing process, NMP is added to reduce the vapor pressure of the ink, and acetone is gradually introduced until a paste-like, homogeneous mixture is achieved. An overview of the ink preparation and printing process is shown in Figure 1, while detailed information on DIW 3D printing is provided in Figure S1 and the diagram of the of rGO/CA ink preparation in Figure S2 of the supplementary materials.

### 2.3. Printing of Electrodes

The DIW printing process was optimized through the careful adjustment of key printing parameters:

- Extrusion Pressure: The pneumatic pressure applied to the syringe was optimized to ensure a steady and controlled ink flow without causing nozzle blockage or excessive material buildup.
- Printing Speed: The printing speed was calibrated to match the extrusion rate, ensuring that each layer was deposited smoothly and without gaps.
- Nozzle Height: The nozzle height was adjusted after each layer to maintain a consistent layer thickness of 150 µm.
- These optimized parameters are presented in Table 1, ensured that each printed electrode had a uniform surface morphology and structural integrity, which is crucial for consistent electrochemical performance.

The prepared ink was loaded into a 10 mL syringe, which was then mounted onto the DIW 3D printer. The extrusion was controlled by a pneumatic system, which allowed for the precise regulation of the ink flow rate. Details of the 3D printing system are provided in Figure S3 of the supplementary materials. The 3D printer was programmed to print electrodes with a square footprint of 10 mm × 10 mm. The thickness of the electrodes was varied by adjusting the number of printed layers, with the layer count ranging from 4 layers to 24 layers.

Each individual layer was printed with a controlled height of 150 µm (0.15 mm), which was achieved by adjusting the nozzle height in the DIW system. The nozzle diameter, printing speed, and extrusion pressure were optimized to ensure uniform layer thickness and smooth surface morphology across the printed layers. The optimization of these parameters was essential to prevent defects such as material sagging or inconsistent layer deposition, which could negatively affect the electrochemical performance of the final electrode.

The number of printed layers directly influenced the overall thickness of the electrode, with thicker electrodes being fabricated by increasing the number of layers. Electrode thicknesses ranged from 600 µm (0.6 mm) for the 4-layer configuration to 3600 µm (3.6 mm) for the 24-layer configuration. The ability to control electrode thickness through layer-by-layer printing allowed for the investigation of how electrode thickness impacts the electrochemical performance, particularly with respect to charge storage capacity and ion diffusion.

**Figure 2:**
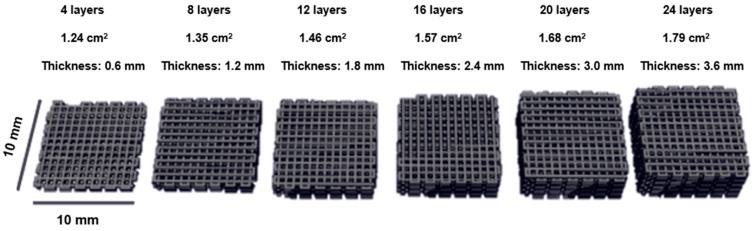
The electrode design, with net shapes measuring 10 × 10 mm^2^, and thicknesses varying from 0.6 mm (4 layers) to 3.0 mm (20 layers).

Computer-aided design (CAD) software version 2.0.2 was used to create electrode models with varying thicknesses. Additionally, CAD provided the geometric surface area of the electrodes, as shown in Figure 2. After printing, the electrodes were left to dry under ambient conditions for 24 h to allow complete solvent evaporation before proceeding with the PANI coating process.

### 2.4. Polyaniline (PANI) Synthesis and Deposition

Polyaniline (PANI) was synthesized through a chemical oxidative polymerization (COP) process using aniline monomer and ammonium persulfate (APS) as the oxidizing agent. The following detailed steps were performed to ensure uniform polymerization and controlled deposition thickness and the list of materials for PANI synthesis is provided in Table S1 of the supplementary materials.

#### 2.4.1. Preparation of Aniline Monomer Solution

First, 0.5 mL of aniline monomer was dissolved in 50 mL of 1 M HCl solution. The acidic environment facilitates the protonation of the aniline, allowing for the subsequent polymerization process. HCl also serves as the doping agent that enables PANI to reach its conductive emeraldine salt form.

#### 2.4.2. Preparation of Redox Initiator

Separately, 1.55 g of ammonium persulfate (APS) was dissolved in 50 mL of 1 M HCl. APS acts as the redox initiator, oxidizing the aniline monomers and driving the polymerization reaction. The APS solution was freshly prepared to avoid degradation and ensure consistent reaction kinetics.

#### 2.4.3. Chemical Oxidative Polymerization (COP) Procedure

**Figure 3:**
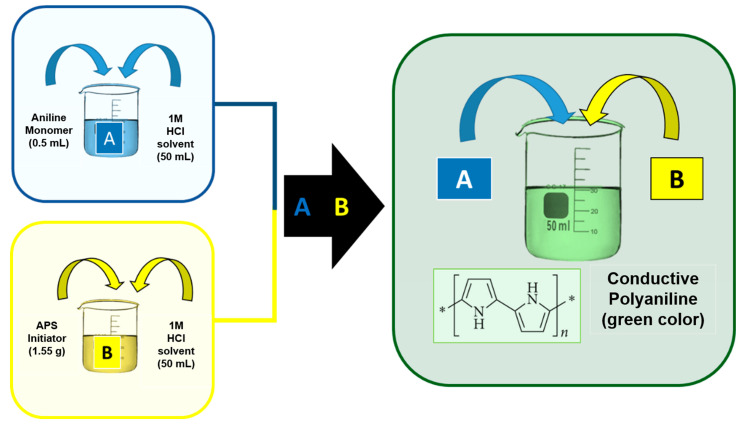
The experimental procedure for preparation of conductive PANI by the in situ chemical oxidative polymerization approach. The asterisk (*) in the PANI formula indicates other molecular structure of PANI.

The aniline monomer solution and APS solution were rapidly mixed under continuous stirring at room temperature (approximately 25 °C), as depicted in the experimental setup shown in Figure 3. Upon mixing, the solution underwent a color change from colorless to green, indicating the formation of the conductive emeraldine salt form of PANI. The polymerization reaction was allowed to proceed for 2 h to ensure complete polymerization.

#### 2.4.4. Precipitation and Purification

After the polymerization reaction, the resulting PANI was precipitated by diluting the reaction mixture with deionized (DI) water, followed by filtering the precipitate. The washing process was repeated three times to remove any unreacted monomers, excess acid, and by-products, ensuring a pure emeraldine salt form of PANI, with an approxi-mate yield of 0.5 g. The filtered PANI was then dissolved in 50 mL of water, creating a 1 wt% PANI colloidal solution. This solution was stirred thoroughly and prepared for the next step, which involves depositing PANI onto 3D-printed rGO electrodes.

#### 2.4.5. Deposition of PANI on 3D-Printed rGO Electrodes

The 3D-printed rGO electrodes (ranging from 4 to 24 layers) were immersed in the PANI solution to deposit a uniform PANI coating. The immersion time was carefully controlled at 10 min per cycle to allow for consistent polymer adsorption on the electrode surface. To achieve different film thicknesses, the PANI coating was applied in multiple cycles (1, 2, 3, 4, and 5 cycles). Between each cycle, the electrodes were dried at room temperature for 60 min to allow for water evaporation and promote uniform film formation.

After each immersion cycle, the electrodes were dried in a controlled environment at room temperature to avoid rapid evaporation, which could lead to cracking or non-uniform deposition.

The thickness of the PANI film was varied by altering the number of deposition cycles (1, 2, 3, 4, and 5 cycles). The film thickness was measured using scanning electron microscopy sourced from Jena, Germany (SEM) cross-sectional imaging after each deposition cycle.

### 2.5. Characterization Techniques

#### 2.5.1. SEM and XRD

This study employed ZEISS Auriga SEM, sourced from Carl Zeiss Microscopy GmbH, Jena, Germany, to produce high-resolution images of a PANI-coated film on a 3D-printed electrode. Using carbon tape, the sample was affixed to the top of a conductive sample holder. The sample was conductive in this case, eliminating the need to coat it with a layer of gold film to enhance image quality. The sample was then loaded into a low vacuum load-lock chamber. A high voltage ranging from 1 kV to 30 kV was applied to operate the SEM.

The phase and structure analysis of the PANI was carried out by X-ray diffraction (XRD; D2 Advance Bruker, sourced from Bruker AXS, Karlsruhe, Germany) with Cu–Kα at λ = 0.15406 nm. The scan range was defined from 10° to 60°, with a time step of 0.5 and a step size of 0.02.

#### 2.5.2. Electrochemical Testing

**Figure 4:**
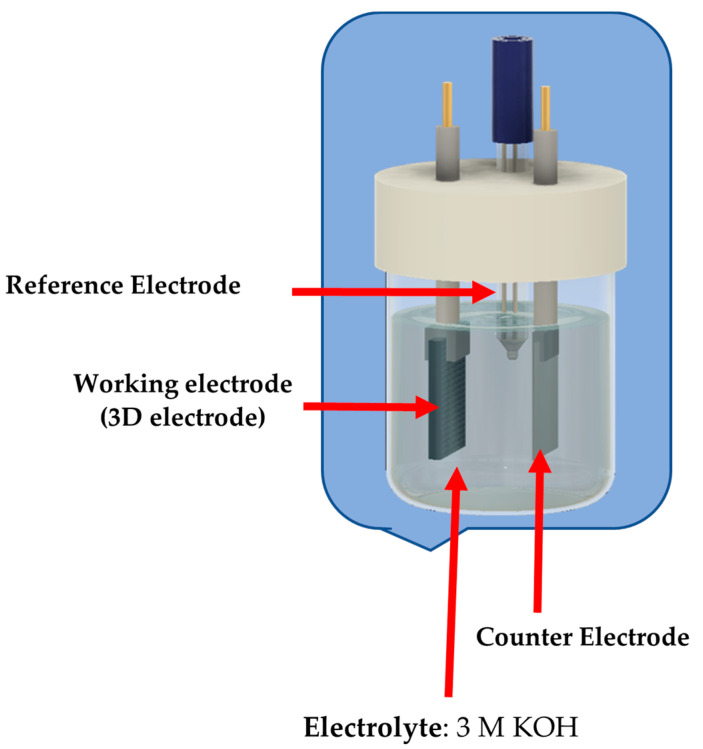
Three-electrode electrochemical cell setup.

The PANI/rGO 3D-printed electrodes were kept soaked in an electrolyte for at least one day, and then the electrodes were ready to be installed in an electrochemical cell. The electrolyte was prepared in an aqueous solution. The 3 M KOH electrolyte was prepared at room temperature. The electrochemical measurements were conducted in a 3-electrode system composed of a platinum counter electrode, an Ag/AgCl reference electrode, and a working electrode, as illustrated in Figure 4 and the experimental setup is shown in Figure S4 of the supplementary materials. The electrochemical cells were connected to the Metrohm Autolab PGSTAT302N, sourced from Metrohm Autolab, Utrecht, the Netherlands, potentiostat/galvanostat system, and Nova 1.11 software was used for operation.

- Cyclic voltammetry

This study measured CV under the conditions of potential windows from −0.9 V to 0.4 V and scan rates of 5, 10, 20, 30, 50, 100, 150, and 200 mV/s. The expected PANI CV curve shows the redox reaction peak, representing the pseudocapacitive nature of the material. For the evaluation of CV, the specific capacitance can be calculated using the following Equations (1) and (2).

Specific capacitance or gravimetric capacitance ($C_{cv})$:

(1) $$C_{cv}=\frac{1}{vm∆V}\int IdV$$

Areal capacitance: the amount of electric charge that can be stored per unit area of one of its electrodes.

(2) $$C_{A}=\frac{1}{vA∆V}\int IdV$$

where ∫IdV is the area of the CV curve, *m* is the mass of active material (g), *A* is the area of the electrode (cm^2^), *v* is the scanning rate (mV/s), and ∆*V* is the voltage window (*V*).

- Galvanostatic charge/discharge

In this study, the GCD was measured with the same potential window as the CV, and the measurement current densities were 0.5, 1.0, 2.0, 3.0, 4.0, and 5.0 A/cm^2^. The GCD charging and discharging are performed by applying current and voltage, resulting in the charging/discharging time. This can be used to evaluate the capacitance with the area ($C_{A}$) of the electrode using Equation (3):

(3) $$C_{A}=\frac{I∆t}{A∆V}$$

where *I* is the discharge current (*A*), *A* is the area of the electrode (cm^2^), ∆*t* is the discharging time (S), and ∆*V* is the voltage window (*V*).

- Electrochemical impedance spectroscopy

In this work, the EIS measurements were performed at the frequency range of 0.01 Hz to 100 kHz at 0.1 V in galvanostatic mode. The equivalent circuits of electrochemical cells were evaluated with Nova 1.11 software.

## 3. Results and Discussion

### 3.1. rGO 3D-Printed Electrode

**Figure 5:**
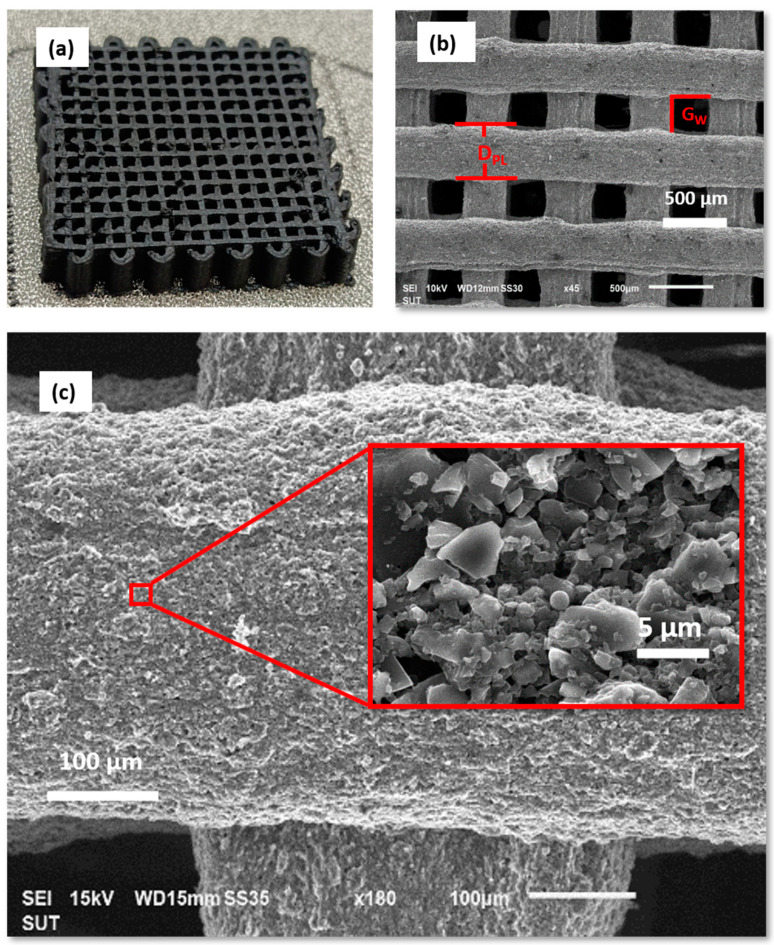
(**a**) Photographs of the 3D-printed electrodes with optimized parameters. (**b**) SEM images of the 3D-printed electrode at 45× magnification. (**c**) SEM image of a 3D-printed electrode, with the inset displaying an enlarged view of a selective region.

The fabrication of the rGO 3D-printed electrode using direct ink writing (DIW) 3D printing was successfully achieved with optimized printing parameters of electrodes with precise dimensions and uniform structure. Figure 5a shows a photograph of the printed electrode on a nickel foam substrate, with designed dimensions of 10 mm × 10 mm × 3 mm (L × W × H), with the printing process completed in approximately 10 minutes.

SEM analysis was used to further characterize the morphology of the printed rGO electrodes. Figure 5b,c display SEM images of the electrode at increasing magnifications (45×, 180×, and 5000×, respectively). The SEM images reveal well-structured rGO lines with clearly defined gaps between adjacent lines, indicating the accuracy of the printing process. The average gap (G_W_) between lines was measured to be 282.92 ± 17.13 µm, while the diameter of the printed lines (D_P_) was found to be 353.15 ± 20.35 µm, as presented in Table 2. The information of the printed lines (D_P_) and the average gap (G_W_) between lines measurement are provided in Tables S2 and S3 of the supplementary materials, respectively. These measurements confirm the consistency of the DIW process in producing uniform electrode structures with minimal deviation from the designed dimensions.

The mass and thickness of the printed electrodes were systematically varied by adjusting the number of printed layers. Table 3 presents the relationship between the number of printed layers, the electrode mass, and the designed electrode height. As expected, increasing the number of layers led to a proportional increase in electrode thickness, ranging from 0.6 mm for the 4-layer electrode to 3.6 mm for the 24-layer electrode. The electrode mass also increased linearly with the number of layers, from 13.0 ± 1.2 mg for the 4-layer electrode to 72.9 ± 3.5 mg for the 24-layer electrode.

These results highlight the controllability of the DIW printing process in producing electrodes with varying thicknesses and masses. The ability to precisely control the electrode dimensions is crucial for optimizing the electrochemical performance of supercapacitors, as the surface area and thickness of the electrode directly influence its charge storage capacity and ion diffusion pathways. This result aligns with findings reported in previous studies, indicating comparable trends in the relationship between electrode dimensions and electrochemical performance [41].

### 3.2. Polyaniline Synthesis

#### 3.2.1. Synthesis and Visual Observations

**Figure 6:**
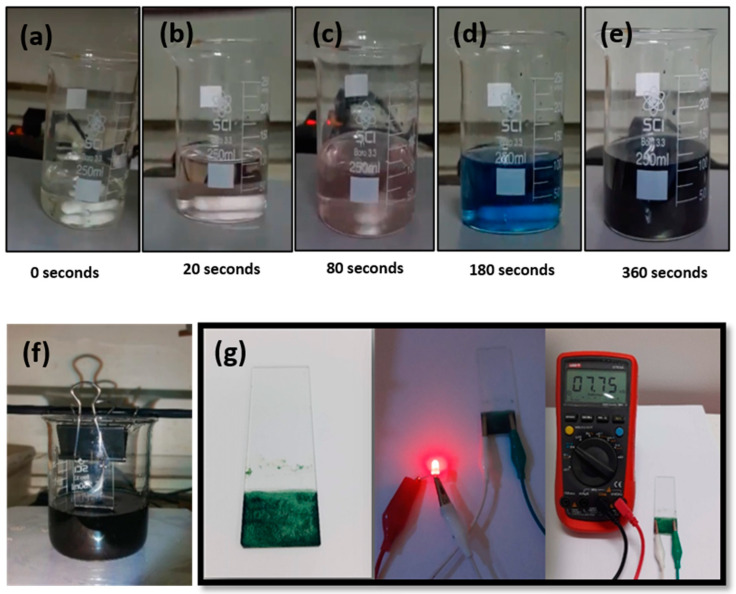
PANI synthesis by chemical oxidative polymerization (COP). Changes in solution color during aniline polymerization at (**a**) 0 s, (**b**) 20 s, (**c**) 80 s, (**d**) 180 s, and (**e**) 360 s; (**f**) PANI deposition on glass slide. (**g**) PANI resistive testing.

Polyaniline (PANI) was synthesized through chemical oxidative polymerization (COP) using aniline monomer and ammonium persulfate as the oxidizing agent. The polymerization process was monitored through the distinct color changes observed over the reaction time, as shown in Figure 6a–e. Initially, the solution was colorless, turning pink after 20 s (Figure 6b), transitioning to a pale blue by 80 s (Figure 6c), and eventually forming a deep green solution after 360 min of reaction (Figure 6e). The final green color confirms the formation of PANI in the emeraldine salt phase, which is known for its high electrical conductivity [42,43,44].

To further test the conductivity of the synthesized PANI, a glass slide was coated with the green PANI solution (Figure 6f), and a simple circuit test was conducted. The coated glass slide was used as a bridge in the circuit to observe whether it could conduct electricity. When placed in the circuit, the coated slide successfully allowed the flow of electric current, illuminating the red LED (Figure 6g). This qualitative experiment indicates that the synthesized PANI is conductive, demonstrating the material’s potential application in supercapacitors and other electronic devices.

#### 3.2.2. X-Ray Diffraction (XRD) Analysis

**Figure 7:**
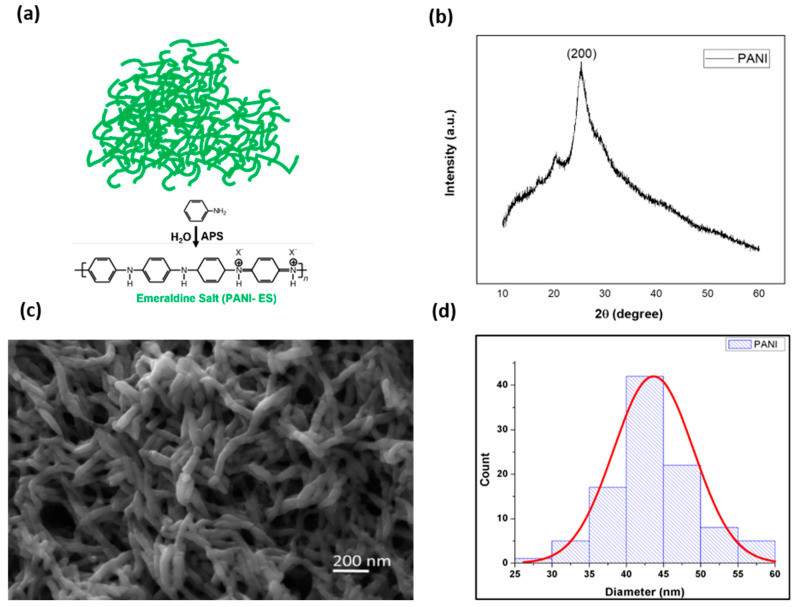
(**a**) Illustration of PANI in emeraldine salt form along with structural formula. (**b**) XRD pattern of the synthesized PANI using the COP method. (**c**) SEM image of the PANI nanofibrous network. (**d**) Histogram of a PANI nanofiber diameter of 46.66 ± 5.50 nm.

The identity of the synthesized PANI was confirmed through X-ray diffraction (XRD) analysis. The illustration of the structure and formula of PANI, shown in Figure 7a, represents the emeraldine salt form of PANI. The XRD pattern, as shown in Figure 7b, displays broad diffraction peaks at 2θ = 20.65° and 25.19°, which correspond to the (020) and (200) crystal planes, respectively. These peaks are characteristic of the amorphous nature of PANI in its emeraldine salt form and are consistent with previous studies [45,46]. The absence of sharp crystalline peaks confirms that the synthesized PANI is largely amorphous, which is favorable for electrochemical applications as amorphous materials tend to exhibit higher ion mobility and more active sites for redox reactions [47].

#### 3.2.3. Scanning Electron Microscopy (SEM) for PANI

Scanning electron microscopy (SEM) was employed to investigate the morphology of the synthesized PANI. The SEM images in Figure 7c reveal a nanofibrous network structure, which is typical of PANI synthesized through the COP method [48]. The fibrous morphology is highly advantageous for supercapacitor applications, as it increases the surface area available for electrolyte interaction and charge storage.

A histogram analysis of the nanofiber diameters, presented in Figure 7d, shows that the average fiber diameter is 46.66 ± 5.50 nm. This nano-scale fibrous structure is expected to significantly enhance the electrochemical performance of the PANI-coated electrodes by providing a larger surface area for redox reactions and improving the accessibility of ions from the electrolyte to the electrode surface [49,50,51,52].

#### 3.2.4. Implications for Electrochemical Performance

The successful synthesis of PANI with a nanofibrous network structure and its confirmed conductivity are crucial for its application in supercapacitor electrodes. The combination of the large surface area provided by the nanofibers and the pseudocapacitive properties of PANI is expected to result in high specific capacitance and enhanced energy storage capability when used in combination with the rGO 3D-printed electrodes.

The synthesized PANI was deposited onto 3D-printed rGO electrodes to further investigate its effect on the electrochemical performance. The fibrous PANI network, when coated on the rGO surface, is expected to improve the electrode/electrolyte interface, facilitating faster ion transport and electron mobility, ultimately leading to higher capacitance and better cycling stability.

### 3.3. PANI/rGO 3D-Printed Electrode

The previously fabricated 3D-printed rGO electrodes, with varying thicknesses, were coated with polyaniline (PANI) using a controlled number of deposition cycles ranging from one to five cycles. The thickness of the PANI coating was directly influenced by the number of deposition cycles, as confirmed through scanning electron microscopy (SEM) analysis.

#### 3.3.1. Surface Morphology and Gap Analysis

**Figure 8:**
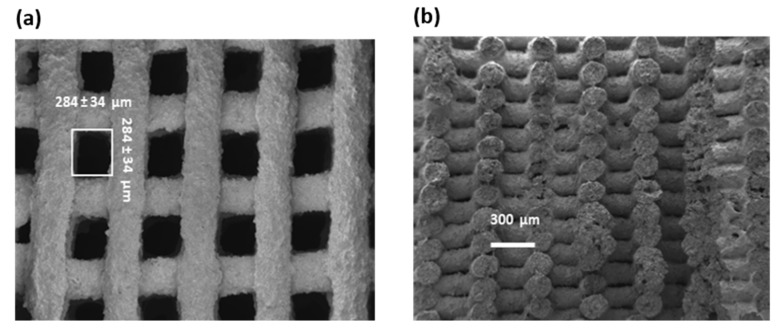
(**a**) Top view SEM image of the PANI/rGO 3D-printed electrode. (**b**) Cross-section of the PANI/rGO 3D-printed electrode.

Figure 8a presents the SEM images of the top view of the PANI/rGO 3D-printed electrodes. A notable feature of the 3D-printed structure is the presence of gaps between the printed rGO lines, which play a crucial role in ion diffusion and electrolyte penetration. The size of these gaps was quantified using image analysis, and the average gap size was determined to be 284 ± 34 µm, as depicted in the histogram in Figure 8b. These gaps are essential for facilitating electrolyte transport within the electrode, which in turn impacts the overall electrochemical performance.

#### 3.3.2. PANI Film Thickness

**Figure 9:**
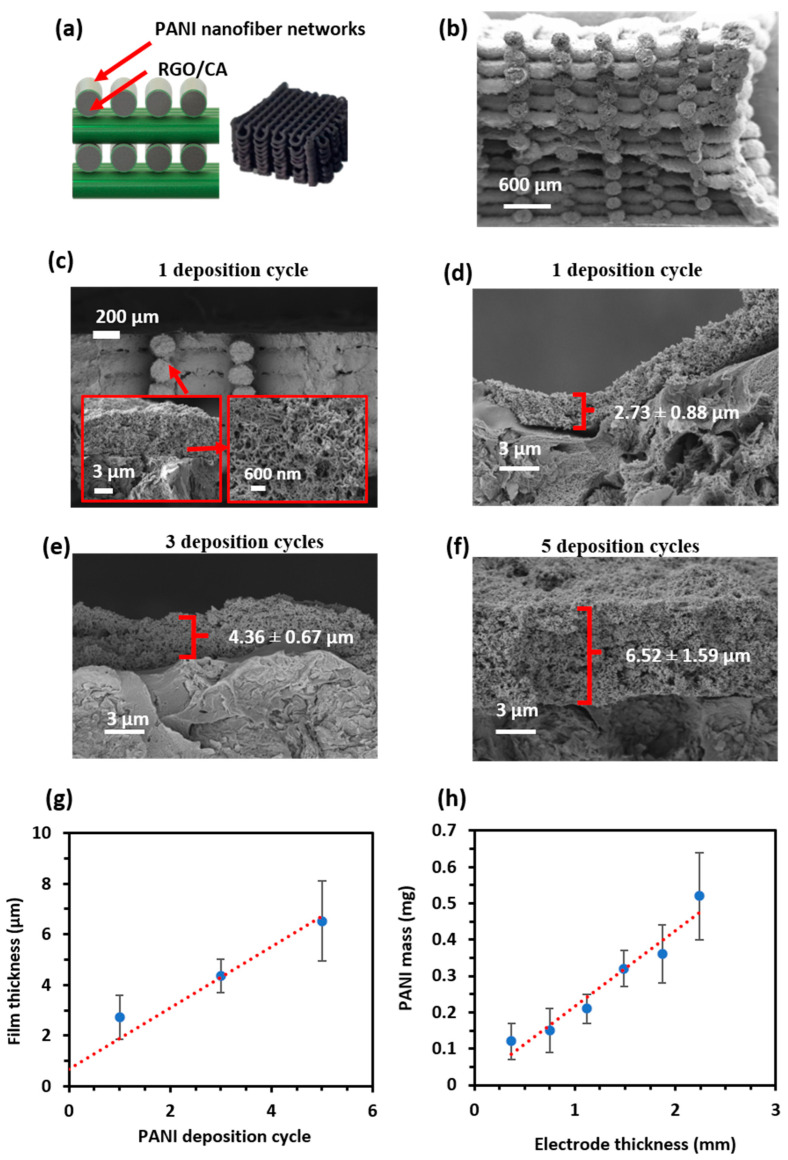
Cross-section of a 24-layer PANI/rGO 3D-printed electrode with different deposition cycles. (**a**) Illustration of PANI/rGO 3D-printed electrode. (**b**) SEM cross-section of a 24-layer 3D-printed electrode. (**c**,**d**) SEM cross-section of a 24-layer rGO 3D-printed electrode with 1 PANI deposition cycle, (**e**) 3 PANI deposition cycles, and (**f**) 5 PANI deposition cycles. (**g**) The graph shows the relationship between the PANI deposition cycle number and film thickness. (**h**) The relationship between the 3D-printed electrode thickness and PANI mass in 1 deposition cycle.

The cross-sectional SEM images, shown in Figure 9a to Figure 9f, reveal the evolution of the PANI film thickness with increasing deposition cycles. A direct correlation was observed between the number of deposition cycles and the thickness of the PANI film, as illustrated in Figure 9g. The detailed measurements of PANI film thickness for one, three, and five deposition cycles are summarized in Table 4. The PANI film thickness increased from 2.73 ± 0.88 µm after one cycle to 6.52 ± 1.59 µm after five cycles, indicating a consistent and controlled growth of the PANI layer with additional deposition cycles.

The uniformity of the PANI coating across the electrode surface was also confirmed through SEM imaging, with minimal variations in thickness across the samples. This controlled deposition process ensured a homogeneous coating, which is critical for enhancing the electrochemical properties of the composite electrode by providing uniform coverage and facilitating effective redox reactions across the entire electrode surface.

#### 3.3.3. Relationship Between Electrode Thickness and PANI Mass

In addition to the number of PANI deposition cycles, the thickness of the 3D-printed electrode was found to have a significant influence on the mass of PANI deposited during each coating cycle. This relationship is shown in Figure 9h and detailed in Table 5. As the electrode thickness increased, the amount of PANI deposited per cycle also increased. For instance, a 3D-printed electrode with a thickness of 0.37 mm had a PANI mass of 0.12 ± 0.05 mg after one deposition cycle, while an electrode with a thickness of 2.24 mm exhibited a PANI mass of 0.52 ± 0.12 mg under the same conditions.

This increase in PANI mass with electrode thickness can be attributed to the larger surface area provided by thicker electrodes, allowing for more PANI to be deposited during each cycle.

#### 3.3.4. Implications for Electrochemical Performance

The variation in PANI film thickness and the amount of PANI deposited on the rGO electrodes are expected to have a significant impact on the electrochemical performance of the composite electrodes. Thicker PANI films provide a greater surface area for redox reactions, which can lead to increased capacitance [53]. However, excessively thick films may introduce diffusion limitations, where the inner layers of the PANI film are less accessible to electrolyte ions, resulting in reduced overall capacitance at higher charge/discharge rates [54,55,56].

Future electrochemical studies, including cyclic voltammetry (CV), galvanostatic charge/discharge (GCD), and electrochemical impedance spectroscopy (EIS), will provide further insights into the trade-offs between film thickness, ion diffusion, and charge storage capacity. Optimizing the balance between electrode thickness and PANI deposition cycles is crucial for achieving high-performance supercapacitors with both high energy density and power density.

### 3.4. Electrochemical Properties 

#### 3.4.1. Areal Capacitance by CV and GCD

**Figure 10:**
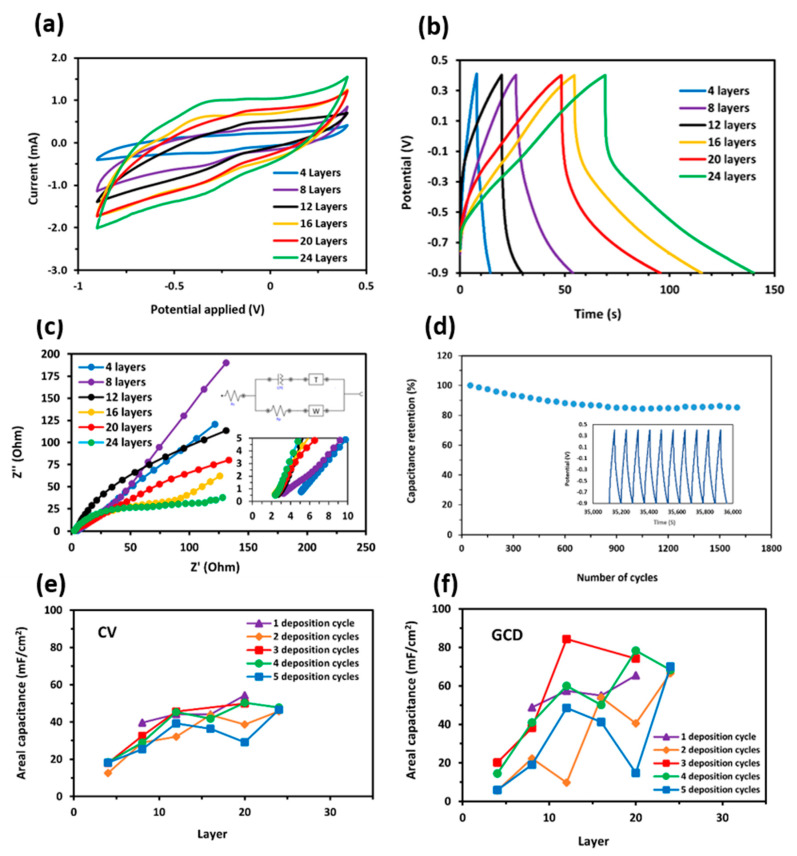
(**a**) The comparison of CV curves of PANI/rGO 3D-printed electrodes of 4 layers to 24 layers with 2 PANI deposition cycles with potential applied of −0.9 V to 0.4 V, at a scan rate of 5 mV/s. (**b**) GCD curve of PANI/rGO 3D-printed electrodes of 4 layers to 24 layers with 2 PANI deposition cycles were measured at a current density of 1 mA/cm^2^ under potential applied of −0.9 V to 0.4 V. (**c**) Nyquist plot of rGO 3D-printed electrodes with 2 PANI deposition cycles with varying printed layers. (**d**) Cycle performance at a current density of 1 mA/cm^2^ for 1600 cycles of 8-layer PANI/rGO 3D-printed electrodes. (**e**) The comparison of CV curves for the areal capacitance of PANI/rGO 3D-printed electrodes with varying printed layers. (**f**) The comparison of GCD curves for the areal capacitance of PANI/rGO 3D-printed electrodes with varying printed layers.

The results from the CV and GCD measurements are presented in Figure 10a,b, respectively. The areal capacitance was found to increase with the thickness of the 3D-printed electrodes with PANI deposition cycles. The CV curves of the PANI/rGO 3D-printed electrodes (Figure 10a) exhibit a nearly rectangular shape, which is indicative of good capacitive behavior and low internal resistance; this is similar to other works [57,58]. The areal capacitance increases steeply for electrodes with 4, 8, and 12 layers, reaching a maximum at 12 layers. Beyond 12 layers, the rate of increase in capacitance begins to diminish, with 16-, 20-, and 24-layer electrodes showing a smaller incremental improvement.

The GCD curves (Figure 10b) further confirm these trends. At a current density of 1 mA/cm^2^, the areal capacitance values follow a similar pattern, with thicker electrodes (12 layers and beyond) showing longer discharge times and higher capacitance values. However, the 20-layer electrode exhibited the highest capacitance value of 54.33 mF/cm^2^ for one PANI deposition cycle by CV, while the 12-layer electrode demonstrated the highest areal capacitance of 84.32 mF/cm^2^ for three PANI deposition cycles, as measured by GCD (Figure 10f).

The increase in areal capacitance with increasing electrode thickness can be attributed to the larger surface area available for charge storage. However, after 12 layers, the diminishing returns observed may result from ion diffusion limitations within the thicker electrodes, which can restrict the accessibility of the entire electrode surface during the charge/discharge process.

#### 3.4.2. Electrochemical Impedance Spectroscopy (EIS)

The EIS measurements provide additional insights into the behavior of the PANI/rGO 3D-printed electrodes. The Nyquist plot (Figure 10c) demonstrates that the resistance of the electrodes increases with electrode thickness. The plot reveals a characteristic semicircle at high frequencies, associated with the charge transfer resistance (R_ct_), and a linear portion at low frequencies, corresponding to the ion diffusion resistance (Warburg impedance). The 24-layer electrode exhibits the largest semicircle, indicating the highest R_ct_, which correlates with its lower electrochemical performance compared to thinner electrodes.

The slope of the Nyquist plot decreases progressively with increasing electrode thickness, further confirming that the resistance is dependent on the number of layers. As the thickness increases, the ion diffusion pathways become longer and more tortuous, leading to increased resistance and reduced ion mobility. This behavior explains the reduced rate of capacitance increase for electrodes with more than 12 layers, as observed in the CV and GCD measurements.

#### 3.4.3. Effect of PANI Deposition Cycles on Capacitance

The number of PANI deposition cycles also significantly impacts the areal capacitance of the electrodes. As shown in Table 6 and Table 7, the areal capacitance increases with the number of PANI deposition cycles up to a certain point, after which it begins to plateau or slightly decrease. This trend is particularly evident for the 12-layer electrodes, where the highest capacitance values were obtained for three PANI deposition cycles: 45.579 mF/cm^2^ (CV) and 84.32 mF/cm^2^ (GCD).

The improvement in capacitance with additional PANI cycles can be attributed to the increased surface area provided by the PANI nanofiber network, which enhances the electrode’s ability to store charge through pseudocapacitance. However, beyond three deposition cycles, the performance gain diminishes, likely due to the excessive thickness of the PANI layer, which can introduce additional resistance and limit ion accessibility to the underlying rGO surface.

#### 3.4.4. Cycling Stability

The cycling stability of the PANI/rGO 3D-printed electrodes was assessed by performing 1600 charge/discharge cycles at a current density of 0.5 mA/cm^2^. The results, shown in Figure 10d, indicate that the electrodes exhibit excellent cycling stability, retaining over 80% of their initial capacitance after 1500 cycles. This high retention rate demonstrates the robustness of the PANI coating and the structural integrity of the 3D-printed rGO electrodes, making them promising candidates for long-term energy storage applications.

#### 3.4.5. Conclusion on Electrochemical Performance

In summary, the electrochemical performance of the PANI/rGO 3D-printed electrodes is highly dependent on both the electrode thickness and the number of PANI deposition cycles. While increasing the thickness of the electrode enhances the areal capacitance by providing more surface area for charge storage, excessively thick electrodes suffer from increased resistance and ion diffusion limitations. The optimal performance was achieved with 12-layer electrodes and three PANI deposition cycles, which exhibited the highest areal capacitance and excellent cycling stability.

Further optimization of the electrode architecture, including the balance between layer thickness and PANI coating, is expected to further enhance the performance of these composite electrodes for practical supercapacitor applications.

Our study on rGO-PANI 3D-printed supercapacitor electrodes explores their potential applications, leveraging 3D printing for on-demand material fabrication. This approach is particularly well suited for planar supercapacitors, enabling the creation of customized flat-pattern electrodes for lightweight and portable devices with limited space.

In the planar configuration, rGO ink is used as the active material, printed directly onto a conductive substrate such as nickel foam or aluminum foil, and subsequently coated with PANI to enhance performance. A gel electrolyte, such as PVA-KOH, is applied uniformly across the electrode surface to ensure ionic conductivity and maintain structural integrity. This setup facilitates efficient operation in compact designs and allows seamless integration into devices with space constraints.

For sandwich-type configurations, the supercapacitor is constructed by stacking two rGO-PANI electrodes, separated by a porous separator (e.g., cellulose paper or a polymer film) soaked in a 3 M KOH aqueous electrolyte. The separator prevents electrical short-circuiting while enabling ionic transport between the electrodes. Current collectors (e.g., stainless steel or aluminum foil with carbon coating) are placed on either side of the electrode-separator assembly to facilitate efficient charge collection. The entire assembly is encapsulated within a rigid casing to ensure structural stability and prevent electrolyte leakage.

This dual adaptability, offering both planar and sandwich-type configurations, highlights the versatility of rGO-PANI materials. The planar design is optimized for lightweight and portable applications, while the sandwich configuration achieves enhanced energy and power densities through efficient stacking. These frameworks demonstrate how rGO-PANI electrodes can inspire innovative designs tailored to diverse energy storage applications.

## 4. Conclusions

This work presents the successful design and fabrication of 3D-printed rGO electrodes enhanced with polyaniline (PANI), achieving notable improvements in supercapacitor electrochemical performance. The unique combination of rGO’s high surface area and conductivity with PANI’s pseudocapacitive properties allowed for significant enhancements in energy storage capabilities. We employed a direct ink writing (DIW) 3D printing process to fabricate electrodes with precisely controlled thickness, ranging from 4 to 24 layers, and optimized the PANI coating through a chemical oxidative polymerization (COP) method with varying deposition cycles.

The results demonstrated that the electrochemical performance of the PANI/rGO 3D-printed electrodes was highly dependent on both the electrode thickness and the number of PANI deposition cycles. Specifically, the 12-layer electrodes coated with three cycles of PANI achieved the highest areal capacitance of 84.32 mF/cm^2^, showcasing excellent charge storage capacity. Beyond 12 layers, the performance gains diminished due to ion diffusion limitations within the thicker electrodes, suggesting that an optimal balance must be struck between electrode thickness and ion transport efficiency.

Additionally, electrochemical impedance spectroscopy (EIS) revealed that the charge transfer resistance (Rct) increased with electrode thickness, further supporting the notion that excessively thick electrodes may hinder the ion diffusion process. Nonetheless, the composite electrodes exhibited good cycling stability, retaining over 80% of their capacitance after 1500 cycles, highlighting the robustness of the PANI coating and the structural integrity of the 3D-printed rGO framework.

This study also demonstrated that increasing the number of PANI deposition cycles improved the electrode’s capacitance by providing additional surface area for redox reactions. However, beyond three cycles, the performance gains diminished, likely due to the introduction of excessive film thickness, which can limit ion accessibility to the inner layers of the PANI coating.

## 5. Future Outlook

The findings of this work offer valuable insights into the design and fabrication of high-performance supercapacitor electrodes. The combination of 3D printing and PANI coating opens new avenues for the development of tunable energy storage devices.

## Figures and Tables

**Table 1:** The printing parameters of the 3D-printed supercapacitor.

Printing Parameters	Values	Description
Layer number	4, 8, 12, 16, 20, and 24 layers	Electrode thicknesses
Layer heights	0.15 mm	This value less than 0.4 mm of nozzle diameter
Printing speed	2 to 40 mm/s	Correlated with air flow pressure
Air flow pressure	137 KPa (20 PSI) to 413 KPa (60 PSI)	Correlated with material viscosity and printing speed
Printing pattern	Net shape (rectilinear pattern)	Simple and higher surface area compared to a solid object shape
Bed temperature	None	The bed temperature function is disabled in this case
Printing perimeter line	1 (line)	Material purged around the electrode before printing

**Table 2:** The observed values of the gap between lines and diameter of lines of the rGO 3D-printed electrode.

Sample Measurement	Average Distance (µm)
Gap (G_W_)	282.92 ± 17.13
Diameter of line (D_PL_)	353.15 ± 20.35

**Table 3:** The comparison of the number of layers in the 3D-printed electrodes with their corresponding heights and masses.

Layer	Electrode Mass (mg)	Designed Electrode Height (mm)	Electrode Area (cm^2^)
4	13.0 ± 1.2	0.6	1.24
8	22.3 ± 1.6	1.2	1.35
12	36.4 ± 3.4	1.8	1.46
16	45.5 ± 2.7	2.4	1.57
20	58.6 ± 1.7	3.0	1.68
24	72.9± 3.5	3.6	1.79

**Table 4:** PANI film thickness with deposition cycle number.

PANI Deposition Cycle	PANI Film Thickness (µm)
1	2.73 ± 0.88
3	4.36 ± 0.67
5	6.52 ± 1.59

**Table 5:** The relationship between 3D-printed electrode thickness and PANI mass in 1 deposition cycle.

Electrode Thickness (mm)	PANI Mass (mg)
0.37	0.12 ± 0.05
0.75	0.15 ± 0.06
1.12	0.21 ± 0.04
1.49	0.32 ± 0.05
1.87	0.36 ± 0.08
2.24	0.52 ± 0.12

**Table 6:** Areal capacitance from CV measurement at scan rate of 5 mV/s, window potential of −0.9 V to 0.4 V, and 3 M KOH.

Printed Layer	Areal Capacitance of 1 PANI Deposition (mF/cm^2^)	Areal Capacitance of 2 PANI Depositions (mF/cm^2^)	Areal Capacitance of 3 PANI Depositions (mF/cm^2^)	Areal Capacitance of 4 PANI Depositions (mF/cm^2^)	Areal Capacitance of 5 PANI Depositions (mF/cm^2^)
4	No	12.488	17.953	18.46	18.064
8	39.635	28.921	32.425	45.382	25.402
12	44.164	32.507	45.579	28.994	39.159
16	43.979	43.816	No	41.743	36.297
20	54.332	38.617	49.908	50.272	29.123
24	No	45.76	No	47.722	46.647

**Table 7:** Areal capacitance from GCD measurement at 1 mA/cm^2^, window potential of −0.9 V to 0.4 V, and 3 M KOH.

**Printed Layer**	**Areal Capacitance of 1 PANI Deposition** **(mF/cm^2^**)	**Areal Capacitance of 2 PANI Depositions** **(mF/cm^2^)**	**Areal Capacitance of 3 PANI Depositions** **(mF/cm^2^)**	**Areal Capacitance of 4 PANI Depositions** **(mF/cm^2^)**	**Areal Capacitance of 5 PANI Depositions (mF/cm^2^)**
4	No	5.65	20.17	14.53	5.83
8	48.85	22.27	38.22	41.01	19.09
12	57.36	9.78	84.32	60.04	48.59
16	55.05	53.928	No	50.11	41.12
20	65.48	40.575	74.138	78.28	14.74
24	No	66.57	No	68.26	69.99

## Data Availability

The data presented in this study are available upon request from the corresponding author.

## Supplementary Materials

The following supporting information can be downloaded at https://www.mdpi.com/article/10.3390/polym16233369/s1: Figure S1: Schematic illustration of the 3D DIW printer. These numbers refer to the following: 1 = 2020 aluminum profile frame constructed, 2 = heat bed or print plate, 3 = 10 mL syringe, 4 = controller board, 5 = switching power supply, 6 = *Z*-axis motor, 7 = *Y*-axis motor, 8 = *X*-axis motor, 9 = LCD display, 10 = air tube, and 11 = air pressure regulator; Figure S2: The diagram of the of rGO/CA ink preparation; Figure S3: (a) Schematic illustration and (b) photograph of the 3D DIW printer. (1) The extruder system, which is a pneumatic dispensing system. (2) The air pressure regulator, which allows selection and control of air pressure from the compressor. (3) The compressor, which provides air pressure to the extruder system; Figure S4: An electrochemical measurement was set up with a 3-electrode computer control system and a Metrohm Autolab PGSTAT302N potentiostat/galvanostat; Table S1: The list of material for PANI synthesis by chemical oxidative polymerization (COP); Table S2: The diameter of the printed line of 50 data points of an rGO 3D-printed electrode was measured using the ImageJ program; Table S3: The gap width measurement of 50 data points of an rGO 3D-printed electrode was made by using the ImageJ program.

## References

[1] Khaled A.A.A.E., Zulkarnain A.N., Norhafezaidi M.S. (2024). Current insights and future prospects of graphene aerogel-enhanced supercapacitors: A systematic review. Heliyon.

[2] Jie Z., Min G., Xi C. (2023). Supercapacitors for renewable energy applications: A review. MNE.

[3] Adedoja O.S., Sadiku E.R., Hamam Y. (2023). An Overview of the Emerging Technologies and Composite Materials for Supercapacitors in Energy Storage Applications. Polymers.

[4] Humaira R.K., Abdul L.A. (2024). Supercapacitors: Overcoming current limitations and charting the course for next-generation energy storage. J. Ind. Eng. Chem..

[5] Abdul G.O., Qaisar A., Ahmed A.M., Mohammad A.A. (2022). Supercapacitors as next generation energy storage devices: Properties and applications. Energy.

[6] Dipanwita M., Mandal M., Bhattacharya S. (2022). Journey from supercapacitors to supercapatteries: Recent advancements in electrochemical energy storage systems. Emerg. Mater..

[7] Yang H., Kannappan S., Pandian A., Jang J.-H., Lee Y.S., Lu W. (2013). Achieving Both High Power and Energy Density in Electrochemical Supercapacitors with Nanoporous Graphene Materials. Nanotechnology.

[8] Kumar N., Kim S.B., Lee S.Y., Park S.J. (2022). Recent Advanced Supercapacitor: A Review of Storage Mechanisms, Electrode Materials, Modification, and Perspectives. Nanomaterials.

[9] Kou L., Huang T., Zheng B. (2014). Coaxial wet-spun yarn supercapacitors for high-energy density and safe wearable electronics. Nat. Commun..

[10] Oktaviardi B.A., Yahdi B.R., Maria U., Dedi F., Ferry I. (2023). Recent progress on reduced graphene oxide and polypyrrole composites for high performance supercapacitors: A review. J. Energy Storage.

[11] Wang Y., Chen Y., Lacey S.D., Xu L., Xie H., Li T., Danner V.A., Hu L. (2017). Reduced graphene oxide film with record-high conductivity and mobility. Mater. Today Phys..

[12] Tene T., Bellucci S., Guevara M., Romero P., Guapi A., Gahramanli L., Straface S., Caputi L.S., Vacacela Gomez C. (2024). Role of Graphene Oxide and Reduced Graphene Oxide in Electric Double-Layer Capacitors: A Systematic Review. Batteries.

[13] Seokhoon C., Changyeon K., Jun M.S., Ho W.J. (2019). Reduced graphene oxide-based materials for electrochemical energy conversion reactions. Carbon Energy.

[14] Du Y., Wang M., Ye X., Liu B., Han L., Jafri S.H.M., Liu W., Zheng X., Ning Y., Li H. (2023). Advances in the Field of Graphene-Based Composites for Energy–Storage Applications. Crystals.

[15] Hegazy H.H., Junaid K., Noshaba S., Eman A.A., Muhammad I.S., Hussein A., Yahia I.S. (2024). 2D-based electrode materials for supercapacitors—Status, challenges, and prospects. RSC Adv..

[16] Qi H., Wang L., Zuo T., Deng S., Li Q., Liu Z., Hu P., He X. (2019). Hollow Structure VS 2 @Reduced Graphene Oxide (RGO) Architecture for Enhanced Sodium-Ion Battery Performance. ChemElectroChem.

[17] Anegbe B., Ifijen I.H., Maliki M. (2024). Graphene oxide synthesis and applications in emerging contaminant removal: A comprehensive review. Env. Sci Eur..

[18] Zhenhu L., Sihan B., Kequan C., Yuping L., Yulin Z., Shuangyi L. (2024). Polyaniline packed activated carbon as pseudocapacitive negative electrodes. J. Chem. Eng..

[19] Wang H., Lin J., Shen Z.X. (2016). Polyaniline(PANi) based Electrode Materials for Energy Storage and Conversion. J. Sci. Adv. Mater. Devices.

[20] Tianyu L., Lauren F., Minghao Y., Hanyu W., Teng Z., Xihong L., Yexiang T., Yat L. (2014). Polyaniline and Polypyrrole Pseudocapacitor Electrodes with Excellent Cycling Stability. Nano Lett..

[21] Beygisangchin M., Abdul Rashid S., Shafie S., Sadrolhosseini A.R., Lim H.N. (2021). Preparations, Properties, and Applications of Polyaniline and Polyaniline Thin Films—A Review. Polymers.

[22] Li Z., Gong L. (2020). Research Progress on Applications of Polyaniline (PANI) for Electrochemical Energy Storage and Conversion. Materials.

[23] Zheng L., Xu Y., Jin D., Xie Y. (2011). Polyaniline-intercalated molybdenum oxide nanocomposites: Simultaneous synthesis and their enhanced application for supercapacitor. Chem. Asian J..

[24] Namsheer K., Chandra S.R. (2021). Conducting polymers: A comprehensive review on recent advances in synthesis, properties and applications. RSC Adv..

[25] Sangeeta R., Mandal U.K., Ashwani K., Yogesh K., Bhawana J. (2021). Enhanced electrochemical performance of hierarchical porous carbon/polyaniline composite for supercapacitor applications. Nano Express.

[26] Pawar D.C., Bagde A.G., Thorat J.P., Lokhande C.D. (2024). Synthesis of reduced graphene oxide (rGO)/polyaniline (PANI) composite electrode for energy storage: Aqueous asymmetric supercapacitor. Eur. Polym. J..

[27] Manoj M., Anilkumar K.M., Jinisha B., Jayalekshmi S. (2017). Polyaniline–Graphene Oxide based ordered nanocomposite electrodes for high-performance supercapacitor applications. J. Mater. Sci. Mater. Electron..

[28] Tadesse M.G., Ahmmed A.S., Lübben J.F. (2024). Review on Conductive Polymer Composites for Supercapacitor Applications. J. Compos. Sci..

[29] Adam M., Daria M., Grażyna G. (2023). Conductive Polymer/Graphene-based Composites for Next Generation Energy Storage and Sensing Applications. ChemElectroChem.

[30] Tiankuo C., Soyeon P., Kun F. (2021). 3D printing-enabled advanced electrode architecture design. Carbon Energy.

[31] Cheng Z., Tianyu L., Fang Q., Wen C., Swetha C., Bin Y., Yu S., Eric B.D., Joshua D.K., Christopher M.S. (2017). 3D printed functional nanomaterials for electrochemical energy storage. Nano Today.

[32] Li Z., Shangwen L., Dayue D., Hanna H., Xiaolong L., Chuhong Z. (2023). Direct Ink Writing 3D Printing for High-Performance Electrochemical Energy Storage Devices: A Minireview. Adv. Sci..

[33] Polychronopoulos N.D., Brouzgou A. (2024). Direct Ink Writing for Electrochemical Device Fabrication: A Review of 3D-Printed Electrodes and Ink Rheology. Catalysts.

[34] Mottaghi M., Pearce J.M. (2024). A Review of 3D Printing Batteries. Batteries.

[35] Solís Pinargote N.W., Smirnov A., Peretyagin N., Seleznev A., Peretyagin P. (2020). Direct Ink Writing Technology (3D Printing) of Graphene-Based Ceramic Nanocomposites: A Review. Nanomaterials.

[36] Rodríguez-Lagar P., Reguera-García A., Llamas-Unzueta R. (2024). Effective, versatile and inexpensive extruder system for direct ink writing of high-viscosity pastes. Int. J. Adv. Manuf. Technol..

[37] Jayakrishna M., Vijay M., Baseem K. (2023). An Overview of Extensive Analysis of 3D Printing Applications in the Manufacturing Sector. J. Eng..

[38] Pelin G., Sonmez M., Pelin C.-E. (2024). The Use of Additive Manufacturing Techniques in the Development of Polymeric Molds: A Review. Polymers.

[39] Foo C.Y., Lim H.N., Mahdi M.A., Wahid M.H., Huang N.M. (2018). Three-Dimensional Printed Electrode and Its Novel Applications in Electronic Devices. Sci. Rep..

[40] Yao B., Chandrasekaran S., Zhang J., Xiao W., Qian F., Zhu C., Duoss E.B., Spadaccini C.M., Worsley M.A., Li Y. (2018). Efficient 3D Printed Pseudocapacitive Electrodes with Ultrahigh MnO_2_ Loading. Joule.

[41] Xue X., Feng L., Ren Q. (2024). Interpenetrated Structures for Enhancing Ion Diffusion Kinetics in Electrochemical Energy Storage Devices. Nano-Micro Lett..

[42] Ali R.E., Milica M.G., Branimir Z.J., Jasmina S.S., Nebojša D.N., Branimir N.G. (2011). Electrochemical synthesis and characterization of polyaniline thin film and polyaniline powder. Prog. Org. Coat..

[43] Prasutiyo Y.J., Manaf A., Hafizah M.A.E. (2020). Synthesis of polyaniline by chemical oxidative polymerization and characteristic of conductivity and reflection for various strong acid dopants. J. Phys. Conf. Ser..

[44] Alok K.Y., Naeem M., Pawan K.K. (2023). Novel synthesis of polyaniline/tellurium (PANI/Te) nanocomposite and its EMI shielding behavior. Mater. Adv..

[45] Vadiraj K.T., Belagali S.L. (2015). Characterization of Polyaniline for Optical and Electrical Properties. IOSR J. Appl. Chem. (IOSR-JAC).

[46] Ambalagi S., Mahalesh D., Nagaraja S., Sannakki B. (2018). Dielectric Properties of PANI/CuO Nanocomposites. IOP Conf. Ser. Mater. Sci. Eng..

[47] Jiawei Z., Yu L., Zhen C., Qian L., Qingguo C., Minghua C. (2023). Amorphous Electrode: From Synthesis to Electrochemical Energy Storage. Energy Environ. Mater..

[48] Abdelaziz R., Mohamed S., Sanjeev M., Sung J.C., John F., Yang D.J. (2008). Polyaniline Nanofiber Synthesis by Co-Use of Ammonium Peroxydisulfate and Sodium Hypochlorite. Chem. Mater..

[49] Sara A., Elzbieta F. (2022). Redox activity from the electrolyte and electrode in electrochemical capacitors. Electrochem. Commun..

[50] Forouzandeh P., Kumaravel V., Pillai S.C. (2020). Electrode Materials for Supercapacitors: A Review of Recent Advances. Catalysts.

[51] Boz E.B., Heijden V.D.M., Jacquemond R.R., Boillat P., Hjelm J., Forner-Cuenca A. (2024). Correlating Electrolyte Infiltration with Accessible Surface Area in Macroporous Electrodes using Neutron Radiography. J. Electrochem. Soc..

[52] An Y.L., Chia C.C., Yi W.L., Peng R.C., Bai C.X. (2020). Improving the Supercapacitor Performance by Dispersing SiO_2_ Microspheres in Electrodes. ACS Omega.

[53] Hacinecipoğlu A.V., Efeoğlu S., Kir B. (2024). Production and applications of lead (II) oxide/poly(aniline-co-thiophene) composite materials for enhanced supercapacitor performance. J. Mater. Sci. Mater. Electron..

[54] Keh C.T., Lei Z., Jiujun Z. (2012). Effects of electrode layer composition/thickness and electrolyte concentration on both specific capacitance and energy density of supercapacitor. Electrochim. Acta..

[55] Xiuxue L., Yubin Z., Wei Y., Guanhua Z., Huai Z., Zhongxue C. (2024). Advances in multi-scale design and fabrication processes for thick electrodes in lithium-ion batteries. Energy Rev..

[56] Ahmed S.E., Joseph H., Johanna R. (2021). Fabrication of Mo1.33CTz (MXene)–cellulose freestanding electrodes for supercapacitor applications. Mater. Adv..

[57] Fei X., Shengxiong Y., Zheye Z., Hongfang L., Junwu X., Lian W., Jun L., Shuai W., Yunqi L. (2015). Scalable Synthesis of Freestanding Sandwich-structured Graphene/Polyaniline/Graphene Nanocomposite Paper for Flexible All-Solid-State Supercapacitor. Sci. Rep..

[58] Ahmed E.-D., Mohamed E.-N., Cheol S.K., Nasser B. (2015). Nitrogen-doped, FeNi alloy nanoparticle-decorated graphene as an efficient and stable electrode for electrochemical supercapacitors in acid medium. Nanoscale Res. Lett..

