# Supplementary material for: Additive Fabrication of Polyaniline and Carbon-Based Composites for Energy Storage
Source: Polymers (Basel). 2024 Nov 29;16(23):3369. doi: 10.3390/polym16233369 (PMC11644309; doi:10.3390/polym16233369)
Supplement: Supplementary file 1 [file polymers-16-03369-s001.zip › polymers-3324526-supplementary.pdf]

Supplementary Information

Additive fabrication of polyaniline and carbon-based composites for energy storage

Niwat Hemha[1], Jessada Khajonrit[2], and Wiwat Nuansing[1,3]

1 School of Physics, Institute of Science Suranaree University of Technology, Nakhon Ratchasima, Thailand; niwat6842@gmail.com
2 Department of Science and Mathematics, Faculty of Science and Health Technology, Kalasin University, Kalasin, Thailand; ex_phys@hotmail.com
3 Center of Excellence on Advanced Functional Materials, Suranaree University of Technology, Nakhon Ratchasima, Thailand; w.nuansing@g.sut.ac.th

* Correspondence: w.nuansing@g.sut.ac.th;

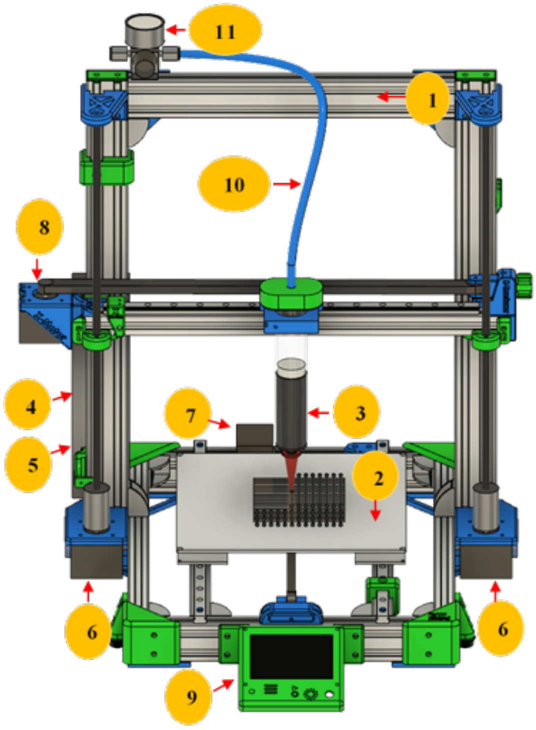

Figure S1. Schematic illustration of of the 3D DIW printer. These numbers refer to the following: 1 = 2020 aluminium profile frame constructed, 2 = heat bed or print plate, 3 = 10 mL syringe, 4 = controller board, 5 = switching power supply, 6 = Z-axis motor, 7 = Y-axis motor, 8 = X-axis motor, 9 = LCD display, 10 air tube, and 11 = Air pressure regulator.

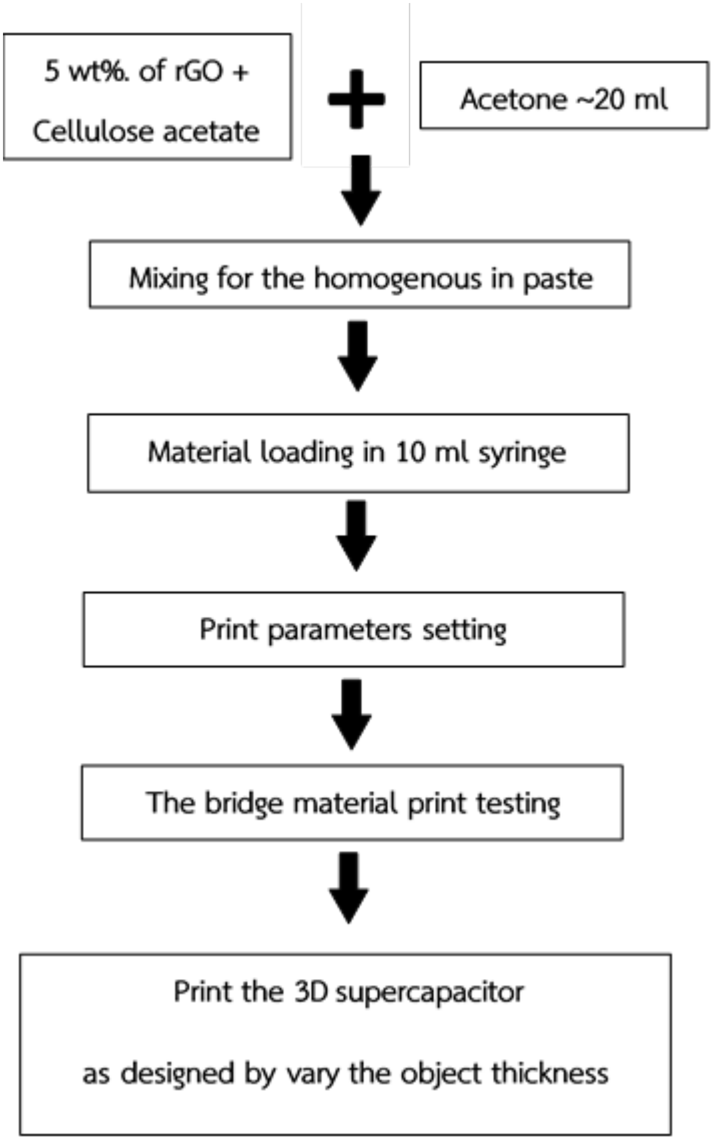

Figure S2. The diagram of the of rGO/CA ink preparation.

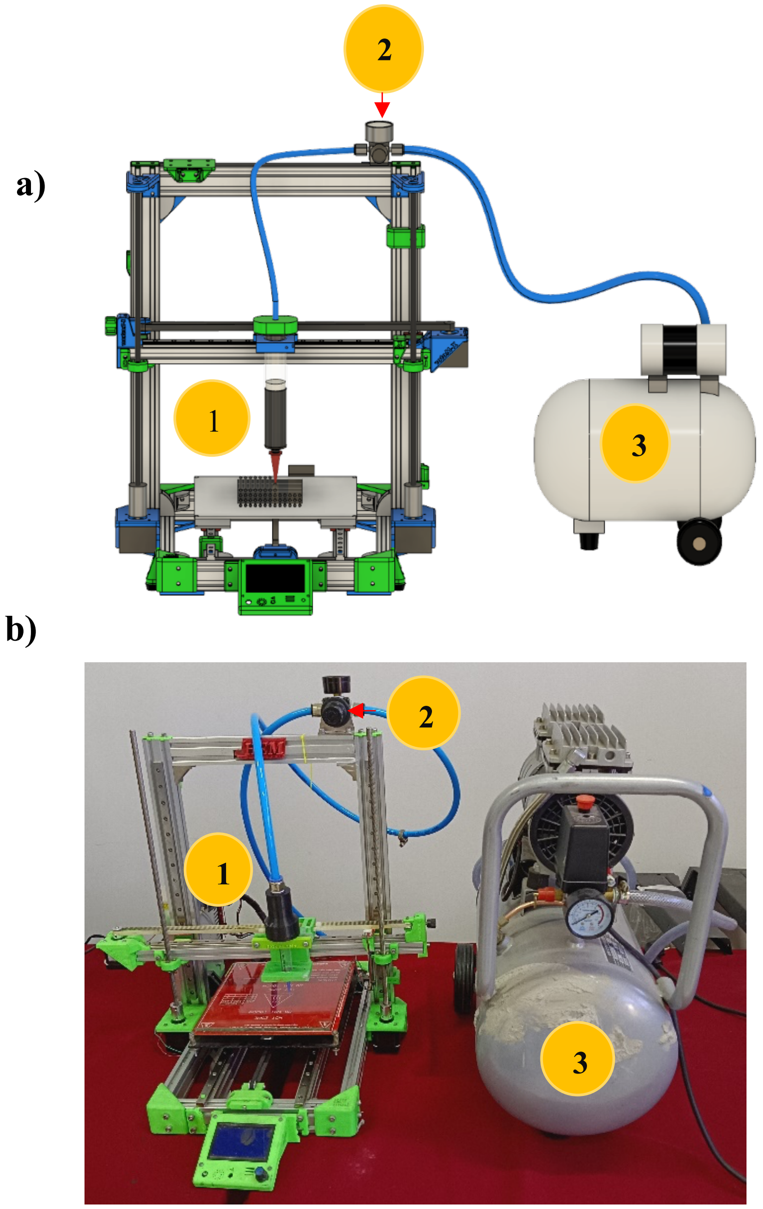

Figure S3. (a) Schematic illustration and (b) photograph of the 3D DIW printer. (1) The extruder system, which is a pneumatic dispensing system. (2) Air pressure regulator, which allows selection and control of air pressure from the compressor. (3) The compressor, which provides air pressure to the extruder system.

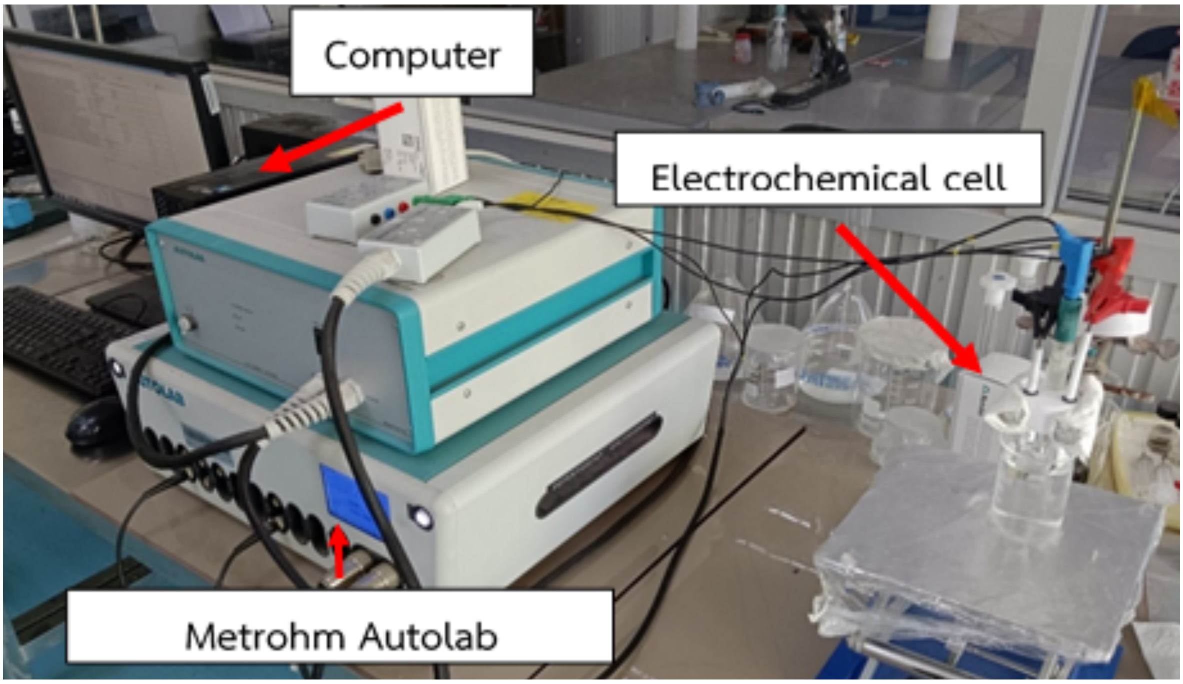

Figure S4. An electrochemical measurement was set up with a 3-electrode computer control system and a Metrohm Autolab PGSTAT302N potentiostat/galvanostat.

Table S1. the list of material for PANI synthesis by chemical oxidative polymerization (COP).

Chemical name	Structure formula	Concentration/ Purity/ Preparation	Supplier	Role
Aniline	$C_6H_5NH_2$	ACS Reagent ≥99.5%. Purified by single or double distillation	Sigma Aldrich	Monomer
Ammonium Persulfate	$(NH_4)_2S_2O_8$	ACS Reagent >98%.	Kemaus	Redox Initiator
Hydrochloric Acid	HCl	1 M (Prepared in lab by dilution of 37% HCl)	Merck (USA, Missouri)	Solvent for monomer, initiator

Table S2. The diameter of the printed line measurement of 50 data points of a rGO 3D-printed electrode was measured using the ImageJ program.

	Area	Mean	Min	Max	Angle	Length
1	770.37	113.945	62	201.8	-91.109	344.509
2	780.247	124.068	37.204	255	-91.459	349.002
3	780.247	129.27	63.25	252.486	-91.464	347.891
4	795.062	123.547	53.5	219.245	-91.975	354.655
5	795.062	125.913	52.581	254.337	-91.611	355.696
6	770.37	113.44	50	222.903	-90.37	344.452
7	800	127.074	63.137	239.883	-90.357	357.044
8	844.444	126.638	69.166	205.083	-90.674	377.804
9	864.198	125.927	63.794	200.605	-90.22	385.929
10	785.185	122.509	60.028	222.423	-90	351.852
11	814.815	133.829	53.41	248.711	-89.768	365.188
12	859.259	119.536	62.628	247.227	-91.325	384.547
13	814.815	127.266	64.147	233.833	-89.768	365.188
14	839.506	133.182	70.889	245.889	-89.094	374.862
15	903.704	137.491	69.19	239.074	-91.787	403.9
16	814.815	131.757	74.925	199.216	-90.813	365.222
17	824.691	131.887	81.63	220.271	-91.614	368.294
18	824.691	126.239	58.243	242.799	-90.23	369.633
19	829.63	126.743	59.497	232.259	-90.115	370.371
20	795.062	122.896	70	212	-90	355.556

21	800	118.744	61.871	237.278	-89.465	356.682
22	824.691	118.046	38.446	231.133	-89.31	368.916
23	795.062	118.304	43.056	248.297	-90.179	355.557
24	720.988	123.179	53.986	237.195	-93.355	322.775
25	760.494	115.215	44.642	241.338	-88.128	340.182
26	804.938	121.329	73.778	220.056	-88.409	360.139
27	839.506	125.797	63.746	253.438	-89.322	375.582
28	809.877	118.556	25	249.699	-93.16	362.774
29	775.309	131.589	58.462	251.846	-91.102	346.731
30	760.494	126.024	60.157	253.039	-91.123	340.065
31	730.864	113.304	59	193	0	326.667
32	809.877	111.655	54.5	166.061	0	361.111
33	711.111	125.84	70	216	0	317.778
34	790.123	115.213	37	224	0	353.333
35	706.173	120.172	63	202.394	-0.807	315.587
36	809.877	117.093	22	174.813	0.705	361.138
37	706.173	120.738	66	176.415	-1.21	315.626
38	795.062	121.76	82.625	194.875	-1.432	355.667
39	735.802	118.054	63	199	0	328.889
40	790.123	110.586	35	180.396	-0.721	353.361
41	750.617	124.68	70.026	208.245	0.379	335.563
42	814.815	119.043	73.634	187.927	0.699	364.472
43	716.049	114.523	24	175.083	-0.398	320.008
44	864.198	128.2	29	245	0.988	386.724
45	725.926	108.619	60	176.849	0.785	324.475
46	829.63	111.865	23	174.784	-0.343	371.118
47	760.494	120.013	37	194.739	-0.749	340.029
48	785.185	131.634	56.304	232.975	-92.175	351.364
49	740.741	113.215	30.195	255	-91.538	331.23
50	720.988	102.098	43	148.379	-1.975	322.414
					**average**	**353.15**
					**sd**	**20.35**

Table S3. The gap width measurement of 50 data points of rGO 3D-printed electrode by using ImageJ program.

	Area	Mean	Min	Max	Angle	Length
1	921.995	14.02	0.25	79	0	300.607
2	931.215	18.851	1.33	53.5	-0.286	303.648
3	875.895	8.589	0.234	90.5	-0.306	283.911

4	866.675	8.804	0	106	-90	280.87
5	958.875	10.718	0.262	117	-90	311.235
6	894.335	11.6	0.729	140	-1.193	291.561
7	885.115	13.797	0.279	193.768	-89.698	288.466
8	903.555	12.583	0	125.716	-91.771	294.675
9	903.555	9.214	0.093	74.75	0	293.016
10	940.435	19.886	1.01	52.525	0.284	306.684
11	931.215	19.409	0.516	177	-88.273	302.263
12	931.215	10.327	0	204	-90	303.644
13	903.555	12.84	0.093	141	-1.181	294.597
14	866.675	9.449	0.129	77	-1.848	282.536
15	857.455	8.124	0	85	-90.623	279.369
16	857.455	12.224	0	131.326	-90.623	279.369
17	885.115	9.946	0.442	74	0.603	288.478
18	885.115	9.524	0	105	-1.206	288.525
19	783.696	9.478	0.238	91	-88.636	255.133
20	940.435	10.441	0	96	0	306.68
21	866.675	16.746	1	169	-88.768	282.454
22	848.236	15.163	0	216	-90	276.316
23	839.016	2.91	0	49	-0.637	273.296
24	866.675	15.617	0	214	-91.848	282.536
25	912.775	12.171	0.531	125	-0.585	297.586
26	820.576	13.559	0	90.795	-88.698	267.275
27	857.455	10.4	0	103	-3.111	279.764
28	894.335	11.247	0	52	0	291.498
29	949.655	12.12	0	84	-90.562	309.731
30	811.356	8.989	0	139	0.659	264.187
31	912.775	17.895	0.153	175	-90.585	297.586
32	949.655	11.914	0.247	93	-0.656	309.231
33	931.215	20.575	0	207	-88.854	303.704
34	866.675	14.837	0	192	-90.616	282.405
35	802.136	6.593	0	65	-0.666	261.151
36	857.455	10.612	0.391	85	-92.49	279.616
37	848.236	9.73	0.967	56.692	-1.259	276.383
38	894.335	15.691	0	112	-90	291.498
39	848.236	16.348	1	112	0	276.316
40	875.895	6.585	0	65	-1.828	285.57
41	820.576	9.1	0	64.089	-89.345	265.706
42	848.236	7.798	0	77	0.63	276.332
43	774.476	15.408	0.5	170	-90	252.024
44	875.895	6.612	0	57	0	283.907
45	820.576	21.871	0	163	-90.326	267.211
46	728.376	7.604	0	63.962	-2.203	237.017

47	783.696	10.888	0	160	-92.058	253.706
48	792.916	10.942	0	73.541	-92.694	258.383
49	848.236	10.273	0	86	-1.888	276.466
50	774.476	10.5	0	126	-90	252.024
					**average**	**282.92**
					**sd**	**17.13**
